# The Importance of the Concentration of Selected Cytokines (IL-6, IL-10, IL-12, IL-15, TNF-α) and Inflammatory Markers (CRP, NLR, PLR, LMR, SII) in Predicting the Course of Rehabilitation for Patients after COVID-19 Infection

**DOI:** 10.3390/biomedicines12092055

**Published:** 2024-09-10

**Authors:** Alicja Mińko, Agnieszka Turoń-Skrzypińska, Aleksandra Rył, Katarzyna Mańkowska, Aneta Cymbaluk-Płoska, Iwona Rotter

**Affiliations:** 1 Department of Medical Rehabilitation and Clinical Physiotherapy, Pomeranian Medical University, 71-210 Szczecin, Poland; agi.skrzypinska@gmail.com (A.T.-S.); aleksandra.ryl@pum.edu.pl (A.R.); iwrot@wp.pl (I.R.); 2 Department of Microbiology, Immunology and Laboratory Medicine, Pomeranian Medical University, 70-111 Szczecin, Poland; katarzyna.mankowska@pum.edu.pl; 3 Department of Reconstructive Surgery and Gynecological Oncology, Pomeranian Medical University, 70-111 Szczecin, Poland; aneta.cymbaluk.ploska@pum.edu.pl

**Keywords:** COVID-19, cytokine, rehabilitation, SARS-CoV-2

## Abstract

Background/Objectives: In the course of COVID-19, there may be an excessive immune response of the body with the release of large amounts of pro-inflammatory cytokines, causing a “cytokine storm”, also known as cytokine release syndrome (CRS). The COVID-19 pandemic has shown how important an integrated approach to health care is, with physiotherapy being one of its fundamental aspects. The aim of this study was to analyze the potential relationship between the level of selected cytokines (IL-6, IL-10, IL-12, IL-15, TNF-α) and inflammatory markers (CRP, NLR, PLR, LMR, SII) and the duration of rehabilitation in patients after COVID-19. Methods: The examined patients participated in a comprehensive rehabilitation program, which included breathing exercises, aerobic training, and strength and endurance training. Peripheral venous blood samples were also collected from each patient. Results: Factors such as gender, smoking status, IL-10, and the presence of pneumonia during infection were significantly associated with the length of rehabilitation after COVID-19. Conclusions: The course of rehabilitation after COVID-19 may depend on many factors, including smoking, the presence of pneumonia due to infection, and some parameters of inflammation.

## 1. Introduction

The SARS-CoV-2 pandemic has significantly impacted the medical and research communities worldwide, with the goal of understanding the complex mechanisms of the body’s response to infection [1,2]. The body’s specific immune response, as a result of which pro-inflammatory cytokines are released, is considered crucial in controlling and treating any viral infection. In the course of COVID-19, there may be an excessive immune response of the body with the release of large amounts of pro-inflammatory cytokines, causing a “cytokine storm”, also known as cytokine release syndrome (CRS) [3,4,5].

The biomarkers detected so far, indicating an increased inflammatory process during SARS-CoV-2 infection, are interleukins (IL), including IL-6, IL-10, IL-12, and IL-15; tumor necrosing factor α (TNF-α); and compounds belonging to routine clinical tests, such as D-dimer and CRP [6,7,8]. Inflammatory markers in the course of SARS-CoV-2 virus infection also include indicators calculated on the basis of blood count results. Numerous studies have shown the usefulness of the indicator calculated on the basis of the neutrophil-to-lymphocyte ratio (NLR), the indicator calculated as the platelet-to-lymphocyte ratio (PLR), and the indicator reflecting the neutrophil-to-platelet number ratio blood (NPR). The systemic inflammation index (SII), calculated as the result of dividing the platelet count by the NLR, also has been proven to be clinically useful [9,10,11].

Differences in the immune profile of COVID-19 patients have been observed. Higher levels of pro-inflammatory cytokines in the blood were associated with a more severe course of the infection. Studies show that the “cytokine storm” was directly correlated with lung damage, multi-organ failure, and unfavorable prognosis for patients with COVID-19 [3,4,5]. One of the most important and multi-directional cytokines is IL-6. Excessive expression of IL-6 plays a pathological role in the development of various inflammatory diseases. Studies show that excessive production of IL-6 could lead to multi-organ failure and contribute to a severe form of COVID-19 [12,13]. IL-10 is a multifunctional cytokine, and its primary function is to limit the inflammatory response. On the other hand, increased expression of IL-10 may act as a factor activating the immune system, thus contributing to the stimulation of the production of other mediators of the cytokine storm [14,15]. The cytokines whose excessive expression may predict a worse prognosis of COVID-19 patients also include IL-12 and IL-15 [16,17,18]. TNF-α, along with other cytokines, is involved in the regulation of inflammatory processes. Serum TNF-α levels have been observed to be elevated in COVID-19 patients and are higher in critically ill patients [13]. CRP is another biomarker with increased level in COVID-19 patients. Elevated CRP levels have been associated with severe infection, increased inflammatory changes in chest CT scans, and increased mortality in patients with COVID-19 [19]. Increased NLR, PLR, and SII were also reported in severe cases of COVID-19, indicating a higher degree of inflammation and a more severe immune response. NLR can also be used to predict hospitalization and mortality risk [20,21,22]. Moreover, PLR was associated with disease progression during hospitalization and longer hospitalization time [21,23]. LMR can be used to predict the risk of death during hospitalization of COVID-19 patients. Higher hospital mortality in COVID-19 patients was associated with lower LMR levels [24,25].

Further research is needed to determine optimal cutoff values for these biomarkers and to explore potential therapeutic strategies targeting these biomarkers. This may help plan the optimal therapeutic process, improving the treatment outcomes for patients with COVID-19, preventing undesirable complications and increased mortality. Based on the current scientific knowledge, there is a clear lack of studies analyzing selected parameters and inflammatory markers that may affect the rehabilitation process in post COVID-19 patients.

A multidisciplinary approach to physiotherapy after COVID-19 is a key element of the effective rehabilitation of patients who have suffered from this disease, especially those who have experienced severe symptoms. The COVID-19 pandemic has shown how important an integrated approach to health care is, with physiotherapy being one of its fundamental aspects [1,26].

The aim of this study was to analyze the potential relationship between the level of selected cytokines (IL-6, IL-10, IL-12, IL-15, TNF-α) and inflammatory markers (CRP, NLR, PLR, LMR, SII) and the duration of rehabilitation in patients after COVID-19. Additionally, the aim of this study was to determine whether there is a relationship between the concentration of these parameters and the time of initiation of rehabilitation after recovery from COVID-19, as well as the functional status of patients. The functional status was assessed based on the results of spirometry and the 6 min walk test.

## 2. Materials and Methods

### 2.1. Patient Qualification for the Study

The study was conducted at the Saint Charles Borromeo Rehabilitation Hospital in Szczecin, Poland, from May 2021 to September 2022. It involved 171 patients staying at the COVID-19 Rehabilitation Department, where medical rehabilitation after SARS-CoV-2 infection was carried out. The qualification of patients for the rehabilitation program, as well as the rehabilitation itself, was carried out in accordance with the guidelines of the National Health Fund (NFZ), indicated in Order No. 42/2021/DSOZ of the President of the National Health Fund of 5 March 2021 [27].

Qualification for rehabilitation was carried out by a physician specializing in therapeutic rehabilitation. The condition for inclusion in the rehabilitation program was the occurrence of complications after COVID-19, assessed on the basis of the following:

- the Post-COVID-19 Functional Status (PCFS) Scale (score 1–4);
- the Medical Research Council (MRC) (score < 5);
- the modified Medical Research Council (mMRC) (score ≥ 1).

The Post-COVID-19 Functional Status Scale is a five-point scale used to identify patients with functional limitations related to various aspects of health after COVID-19 [28]. The Medical Research Council is a scale used to test muscle strength ranging from 0 to 5, where 0 means no muscle tension and 5 means normal muscle strength [29]. The modified Medical Research Council is a five-point scale assessing the severity of shortness of breath, where 0 means shortness of breath only during physical exercise and 4 means shortness of breath that prevents leaving home [30].

The study included adults whose diagnosis of COVID-19 was confirmed based on a positive polymerase chain reaction (PCR) test for SARS-CoV-2. The patients participating in the study had their first COVID-19 infection and had not previously participated in other post-COVID-19 rehabilitation. Inclusion criteria included a period of not exceeding 12 months from the end of COVID-19 treatment and consent to collecting biological material (blood). The end of COVID-19 treatment was defined as the date of completion of home isolation, discharge from hospital, or solitary confinement. The required diagnostic test for qualification for rehabilitation was also a chest X-ray with description, taken after completion of treatment in the acute phase of the disease. Patients under 18 years of age, patients who did not consent to the collection of biological material, and patients with diseases that prevented them from consenting to the study or understanding its nature and conditions of participation in it were excluded from the study. Ultimately, taking into account all inclusion and exclusion criteria, 167 patients participated in the study.

Each patient gave written informed consent to participate in this study and to use data from their medical records. Every effort has been made to protect the privacy and anonymity of patients. The study was conducted in accordance with the current version of the Declaration of Helsinki. Approval to conduct the study was obtained from the Bioethics Committee of the Pomeranian Medical University in Szczecin (decision no. KB-0012/15/2021).

### 2.2. Study Process

**Figure 1:**
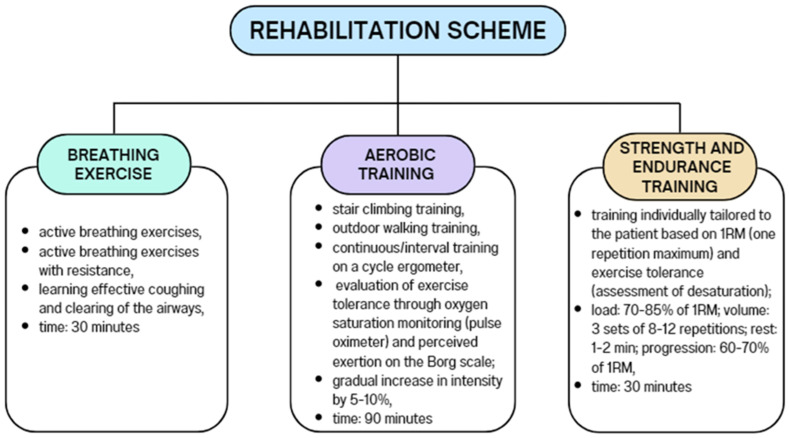
Rehabilitation scheme.

The examined patients participated in a comprehensive rehabilitation program, which included breathing exercises, aerobic training, and strength and endurance training. A detailed description of the procedures performed is presented in Figure 1. Rehabilitation took place six times a week, from Monday to Saturday. The minimum rehabilitation period that the patient had to complete was 2 weeks. The decision to extend rehabilitation (up to a maximum of 6 weeks) was made by the attending physician based on a comparison of the current test results with the results performed before the start of rehabilitation. This included an exercise test (6 min walk test) with an assessment of exercise tolerance (Borg scale), an assessment of the severity of shortness of breath (the mMRC scale), and a spirometric assessment of respiratory function. Throughout the entire rehabilitation period, the patient was under medical, nursing, and physiotherapeutic care.

Spirometry tests were performed using the BTL-08 Spiro Pro spirometer (BTL Industries, Newcastle-under-Lyme, UK). The following parameters were determined: forced expiratory volume in the first second (FEV 1), forced vital capacity (FVC), and FEV1/FVC ratio. Results were expressed as a percentage of the patient’s predicted normal values, which were calculated automatically based on age, gender, height, weight, and ethnicity. The FEV1/FVC ratio is shown as an absolute value. For the interpretation of spirometry data, ECCS/ERS 1993 reference values were used. All spirometric measurements were performed in accordance with standard American Thoracic Society (ATS) and European Respiratory Society (ERS) recommendations [31].

The 6MWT was conducted according to the American Thoracic and European Respiratory Society standards [32]. The 6MWT measurement was performed along a straight, 30 m long paved corridor. The distance the patient traveled in 6 min was measured. Results are expressed as a percentage of predicted normal values for each patient. The predicted values of 6MWD were calculated based on the following formulas:

6MWT [m] = (7.57 × height [cm]) − (5.02 × age [years]) − (1.76 × weight [kg]) − 309 (for men),

6MWT [m] = (2.11 × height [cm]) − (2.29 × weight [kg]) − (5.78 × age [years]) + 667 (for women).

On the day of commencement of rehabilitation, an interview was conducted with each subject to obtain sociodemographic data. Information on the course of the disease and treatment, as well as comorbidities, was obtained from medical records. Peripheral venous blood samples were also collected from each patient.

### 2.3. Calculation of Inflammation Severity Indicators Based on Blood Counts

Based on the blood counts performed at the beginning of rehabilitation, the following indicators of inflammation severity were calculated:

- neutrophil-to-lymphocyte ratio (neutrophil/lymphocyte ratio—NLR);
- ratio of the platelets to the lymphocytes (platelet/lymphocyte ratio—PLR);
- ratio of the lymphocytes to the monocytes (lymphocyte/monocyte ratio—LMR);
- ratio of the product of the platelets and the neutrophils to the number of lymphocytes (systemic inflammation index—SII) [33,34].

The CRP level [mg/L] was also determined, the laboratory norm of which is 0.00–5.00 mg/mL [35].

### 2.4. ELISA Tests

Blood was collected into test tubes containing ethylenediaminetetraacetic acid (EDTA). To obtain plasma, the tubes were centrifuged, then frozen and stored at −80 °C until laboratory analyses were performed.

Before laboratory analyses, the samples were thawed at room temperature. All determinations were performed using enzyme-linked immunosorbent assay (ELISA) from Sun Red Biotechnology Company, Shanghai, China, according to the manufacturer’s instructions. First, plates containing standards were prepared. Then, biotin-labeled antibodies, test material, and streptavidin were added. The volume of the tested material and reagents depended on the parameter being determined. The prepared plates were incubated for 60 min at 37 °C and then washed five times with a washing buffer. Then, chromogen A and B were added, the mixture was incubated for 10 min at 37 °C, and the inhibitor solution was applied. Absorbance was measured at a wavelength of 450 nm, and Envision^®^ software (EnVision 2104 Multilabel Plate Reader; PerkinElmer, Waltham, MA, USA) was used for analysis based on a linear curve. The following levels were determined as follows: IL-6 [pg/mL], IL-10 [pg/mL], IL-12 [pg/mL], IL-15 [pg/mL], TNF-α [pg/mL].

### 2.5. Statistical Analyses

Statistical analysis was performed using Statistica 13.1 software (StatSoft, Inc., Tulsa, OK, USA). Descriptive statistics, including the number of patients, patient percentages, mean, and standard deviation, were used to characterize the study group. The normality of distribution was assessed using the Shapiro–Wilk test. Step-by-step multiple regression analysis was performed to develop a model to identify independent factors influencing the studied parameters. The best modeling was selected. Only the regression solution is presented, and the individual steps are described in the text. A statistical significance was attributed to results where the *p*-value was lower than 0.05.

## 3. Results

Detailed information regarding the characteristics of the study group is presented in Table 1.

Step-by-step regression analysis was performed to examine predictors that may influence the factors under study. Due to the large number of independent variables included in the analyses, only the initial regression model is presented, and all statistically significant results are described in the text.

Table 2 shows the step-by-step regression analysis with the dependent variable (rehabilitation time) along with all the independent variables. It was shown that gender (β = −0.27) and pneumonia (β = 0.359) during COVID-19 infection were significantly associated with the dependent variable. In subsequent traffic jams after removal of SII and age, the following were significantly associated with rehabilitation time: gender (β = −0.27), pneumonia (β = 0.359), and IL-10 (β = −0.21). In the next steps: LMR, NLR, and PLR were rejected. Statistically significant variables included gender (β = −0.28), pneumonia (β = 0.363), IL-10 (β = −0.21), and smoking status (β = −0.20). After removing the next factor, namely, IL-6, statistically significant results were gender (β = −0.27), pneumonia (β = 0.357), and IL-10 (β = −0.21). In the next steps, the following were rejected: IL-15, BMI, and IL-12. At this stage, significance was demonstrated with gender (β = −0.26), pneumonia (β = 0.345), and smoking status (β = −0.20). This relationship persisted in subsequent steps until all other variables were rejected. Ultimately, three statistically significant dependencies remained: gender (β = −0.27), pneumonia (β = 0.410), and smoking status (β = −0.21).

Table 3 presents a step-by-step regression analysis with the dependent variable (the period of time from the end of COVID-19 treatment to the start of rehabilitation) along with all independent variables. It was shown that age was significantly related to the dependent variable (β = 0.336). The significance also remained after the rejection of variables such as SII and gender. After removing the PLR variable, the analysis showed significance with age (β = 0.337) and NLR (β = −0.31). In the next steps, IL-15, IL-6, pneumonia, and IL-12 were rejected. In addition to age and NLR, IL-10 (β = 0.212) turned out to be an important factor. In the next steps, CRP, LMR, BMI, and diabetes were rejected. Ultimately, three statistically significant dependencies remained: age (β = 0.273), NLR (β = −0.26), and IL-10 (β = 0.207).

Table 4 presents a step-by-step regression analysis with the dependent variable (CRP) along with all the independent variables. After eliminating the following variables: SII, BMI, diabetes, smoking status, PLR, and NLR, statistical significance with LMR was demonstrated (β = −0.22). After eliminating the remaining factors (sex, pneumonia, age), there was still a significant relationship with LMR (β = −0.24).

Table 5 presents a step-by-step regression analysis with the dependent variable, namely, NLR, along with all independent variables. After eliminating variables such as gender, FVC, and 6MWT, statistical significance was demonstrated with FEV1 (β = −0.29). The significance of this independent variable remained until the end of the analysis. In subsequent steps, the following items were excluded: pneumonia, age, diabetes, smoking status, BMI, and FEV1/FVC. Only one variable showed statistical significance at the end of the analysis—FEV1 (β = −0.26).

Table 6 presents a step-by-step regression analysis with the dependent variable, namely, SII, along with all independent variables. After eliminating variables such as gender, FVC, 6MWT, age, diabetes, and pneumonia, statistical significance was demonstrated with FEV1 (β = −0.25). In the next steps, BMI and smoking status were rejected. After discarding FEV1/FVC, there was no significant association with FEV1.

## 4. Discussion

To our knowledge, this study is the first to examine the impact of selected cytokines and inflammatory markers on the duration of rehabilitation after COVID-19. This study may significantly influence the development of innovative diagnostic and physiotherapeutic strategies in the treatment and rehabilitation of patients infected with SARS-CoV-2. Taking into account the role of inflammation in the pathomechanism of COVID-19, our study focused on analyzing the impact of selected cytokines and inflammatory indicators on the rehabilitation period of patients recovering after infection. Existing research on the enhanced inflammatory process during SARS-CoV-2 infection usually focuses on its relationship with the severity of COVID-19 and the mortality rate.

Numerous studies prove that inflammation is closely related to the severity of COVID-19 [3,5]. It has been shown that some parameters related to inflammation, such as TNFα, IL-6, interleukin-8 (IL-8), interleukin-10 (IL-10), CRP, white blood cell count (WBC), lymphocyte count (LC), and the number of neutrophils (NC) are correlated with the severity of COVID-19 [36,37,38]. The inflammatory process can increase inflammation in the muscles. An increase in the concentration of C-reactive protein and an increased level of other cytokines, such as IL-1, IL-6 and TNF-α, contributes to the proteolysis of muscle fibers, activation of fibroblasts, and blocking of progenitor cells responsible for the formation of new muscle fibers [39,40]. These phenomena explain why physical and respiratory recovery after COVID-19 infection is so difficult.

The results of our research indicate a significant relationship between rehabilitation time and IL-10. However, after taking into account potential confounding factors, it turned out that better predictors of the length of rehabilitation after COVID-19 are gender, the presence of pneumonia in the course of infection, and smoking status. IL-10 is an anti-inflammatory cytokine that plays a role in regulating the inflammatory response [41]. Lower IL-10 levels may suggest insufficient suppression of the inflammatory response, which may lead to a longer time needed for rehabilitation. These results highlight the complex relationship between inflammation and the rehabilitation process. In addition, the influence of other factors may also have an impact on predicting the length of rehabilitation. According to our research, such factors include gender, smoking status, and the presence of pneumonia during COVID-19 infection. Further research is needed to better understand the mechanisms underlying these associations and their potential clinical implications.

Our study found significant associations between inflammatory markers and inflammation as measured by CRP. The increase in CRP was associated with an increase in LMR, suggesting increased inflammation. These results indicate that an increase in CRP, as an inflammatory marker, is associated with an increase in LMR, which reflects the severity of the inflammatory process. These associations highlight the importance of monitoring these indicators in the assessment and treatment of inflammatory processes in patients.

Our research showed significant associations between inflammatory markers such as NLR and IL-10 and the period of time from the end of COVID-19 treatment to the start of rehabilitation. The results suggest that the time of starting rehabilitation after completing COVID-19 treatment may be related to the indicated inflammatory parameters. Moreover, our research also showed a significant influence of the patient’s age.

Our analysis shows that inflammatory indicators such as NLR and SII may be related to forced expiratory volume in one second (FEV1) in patients after COVID-19. NLR and SII values, which reflect an increased inflammatory response, may affect lung function parameters such as FEV1. These observations are consistent with previous reports suggesting that inflammation may have a significant impact on respiratory performance. A study by Sirayder et al. [42] shows the correlation between CRP and lactate dehydrogenase (LDH) values in patients with COVID-19 and FVC. In the group of patients with COVID-19, a significant relationship was found between CRP and FVC. Lactate dehydrogenase levels were negatively correlated with FVC. These studies suggest that CRP and lactate dehydrogenase values are associated with inflammation, which may have a significant impact on respiratory function in patients after COVID-19.

The observed associations between inflammatory markers and lung function underscore the importance of monitoring inflammation in patients undergoing rehabilitation. Managing inflammation can be crucial to improving patients’ respiratory function and overall physical condition.

The observed results between inflammatory markers and lung function highlight the importance of monitoring inflammation in patients undergoing rehabilitation. Future research should focus on the mechanisms underlying these associations, which may help identify vulnerable individuals requiring specialized care, as well as develop effective intervention strategies.

An important aspect to consider is the polarization states of immune cells, particularly the dual influence of M1 and M2 macrophage polarization on the progression of disease. Macrophages can be polarized towards a pro-inflammatory M1 phenotype by IFN-γ and lipopolysaccharide, or towards an anti-inflammatory M2 phenotype by IL-4 or other factors [43]. Viral infections can induce M1 macrophage polarization, which plays a crucial role in combating the virus due to the release of pro-inflammatory cytokines. These cytokines attract a significant number of immune cells, such as neutrophils and dendritic cells, to the site of infection, thereby generating antiviral immunity. However, in the case of SARS-CoV-2 infection, macrophages appear to contribute to an excessive inflammatory response, exacerbating the pathogenesis associated with SARS-CoV-2 [44,45]. Different macrophage polarization states can either protect the body during recovery or cause harm due to an excessive immune response. There is evidence suggesting that M1 macrophages enhance and spread the SARS-CoV-2 virus, while M2 macrophages degrade and limit its spread [46]. Understanding this aspect is crucial for grasping the mechanisms of cytokines and inflammatory markers. Moreover, understanding the complex interaction between macrophages and SARS-CoV-2 could lead to the discovery of new therapeutic targets against this viral infection.

There are some limitations to this study. First of all, participants were recruited from a single research center. A second limitation is the lack of data on inflammatory markers from the early stage of infection. The limitations of the study also include the lack of information regarding the vaccination status of patients, as well as the type of vaccines, the number of doses, and the time of the last dose in relation to the diagnosis of COVID-19, which could have a possible impact on the results.

## 5. Conclusions

The course of rehabilitation after COVID-19 may depend on many factors, including smoking, the presence of pneumonia due to infection, and some parameters of inflammation. Indicators calculated on the basis of a complete blood count are a relatively cheap and widely available test that can provide important information related to the course of the inflammatory process in patients after COVID-19.

## Figures and Tables

**Table 1:** Characteristics of the study group.

Variable		n	%
Sex	Female	91	53.2
	Male	76	46.8
Age	<60 years	46	26.9
	>60 years	121	73.1
Nutritional status (BMI)	18.5–24.99 (norm)	36	21.1
	25.0–29.9 (overweight)	60	35.1
	30.0–34.99 (1st degree obesity)	48	28.1
	35.0–39.99 (2nd degree obesity)	18	10.5
	Over 40 (3rd degree obesity)	5	2.9
Hospitalization	Yes	117	68.4
	No	44	25.7
Length of hospitalization	1–5 days	5	2.9
	6–10 days	18	7.6
	11–15 days	31	18.1
	16–20 days	18	10.5
	More than 20 days	50	29.2
Pneumonia during COVID-19 infection	Yes	128	74.8
	No	34	19.9
Oxygen therapy during hospitalization	Yes	106	61.9
	No	38	22.2
The duration of rehabilitation	2–3 weeks	75	43.8
	3–4 weeks	23	13.5
	4–5 weeks	16	9.4
	5–6 weeks	51	29.8
Comorbidities	Without comorbidities	37	22.2
	≥1 comorbid disease	130	77.8
Type of comorbid disease	Diabetes	50	29.2
	Hypertension	113	66.1
	Asthma	19	11.1
	COPD	9	5.3
Smoking status	Yes	15	8.8
	No	146	85.4
Concentration of cytokines	M ± SD		
	IL-6 (pg/mL)	285.51 ± 288.98	
	IL-10 (pg/mL)	491.95 ± 554.83	
	IL-12 (pg/mL)	105.97 ± 110.19	
	IL-15 (pg/mL)	96.01 ± 83.99	
	TNF-α (pg/mL)	148.84 ± 179.19	
Inflammation severity index value	NLR	2.33 ± 1.99	
	PLR	138.99 ± 115.95	
	LMR	3.57 ± 1.29	
	SII	635.73 ± 810.18	
	CRP	5.89 ± 14.92	

Legend: n—number of patients; BMI—Body Mass Index; M—mean; SD—standard deviation; IL-6—interleukin-6; IL-10—interleukin-10; IL-12—interleukin-12; IL-15—interleukin-15; TNF-α—tumor necrosis factor α; NLR—neutrophil/lymphocyte ratio; PLR—platelet/lymphocyte ratio; LMR—lymphocyte/monocyte ratio; SII—The Systemic Immune-Inflammation Index; CRP—C-reactive protein.

**Table 2:** Step-by-step regression results with rehabilitation time as the dependent variable.

	Rehabilitation Time		
Independent Variables	Beta Significance	Independent Variables	Beta Significance
Sex	−0.27 *	IL-15	0.081
Age	0.004	CRP	0.129
BMI	0.09	LMR	−0.01
Diabetes	0.156	NLR	−0.03
Pneumonia	0.359 *	PLR	0.053
IL-6	−0.07	SII	0.00
IL-10	−0.21	Smoking status	−0.19
IL-12	0.104	TNF-α	−0.13

Legend: IL-6—interleukin-6; IL-10—interleukin-10; IL-12—interleukin-12; IL-15—interleukin-15; NLR—neutrophil/lymphocyte ratio; PLR—platelet/lymphocyte ratio; LMR—lymphocyte/monocyte ratio; SII—The Systemic Immune-Inflammation Index; CRP—C-reactive protein; TNF-α—tumor necrosis factor α; BMI—Body Mass Index; * statistical significance <0.05.

**Table 3:** Step-by-step regression results with the time period from the end of COVID-19 treatment to the start of rehabilitation as the dependent variable.

	The Period of Time from the End of COVID-19 Treatment to the Start of Rehabilitation		
Independent Variables	Beta Significance	Independent Variables	Beta Significance
Sex	0.007	IL-12	0.089
Age	0.336 *	IL-15	0.037
BMI	0.125	CRP	−0.11
Diabetes	−0.16	LMR	−0.13
Pneumonia	−0.07	NLR	−0.29
IL-6	−0.05	PLR	−0.02
IL-10	0.178	SII	0.00

Legend: IL-6—interleukin-6; IL-10—interleukin-10; IL-12—interleukin-12; IL-15—interleukin-15; NLR—neutrophil/lymphocyte ratio; PLR—platelet/lymphocyte ratio; LMR—lymphocyte/monocyte ratio; SII—The Systemic Immune-Inflammation Index; CRP—C-reactive protein; BMI—Body Mass Index; * statistical significance <0.05.

**Table 4:** Step-by-step regression results with the dependent variable—CRP.

	CRP		
Independent Variables	Beta Significance	Independent Variables	Beta Significance
Sex	−0.07	LMR	−0.19
Age	0.097	NLR	0.090
BMI	−0.03	PLR	−0.16
Diabetes	0.036	SII	0.106
Pneumonia	0.90	Smoking status	−0.04

Legend: NLR—neutrophil/lymphocyte ratio; PLR—platelet/lymphocyte ratio; LMR—lymphocyte/monocyte ratio; SII—The Systemic Immune-Inflammation Index; CRP—C-reactive protein; BMI—Body Mass Index.

**Table 5:** Step-by-step regression results with the dependent variable—NLR.

	NLR		
Independent Variables	Beta Significance	Independent Variables	Beta Significance
Sex	−0.01	FVC	0.085
Age	0.043	FEV1/FVC	0.216
BMI	−0.12	FEV1	−0.38
Diabetes	0.071	6MWT (%)	−0.02
Pneumonia	0.038	Smoking status	−0.09

Legend: NLR—neutrophil/lymphocyte ratio; 6MWT—6 min walk test; FVC—forced vital capacity; FEV1—forced expiratory volume in the first second; BMI—Body Mass Index.

**Table 6:** Step-by-step regression results with the dependent variable—SII.

	SII		
Independent Variables	Beta Significance	Independent Variables	Beta Significance
Sex	0.020	FVC	0.239
Age	−0.05	FEV1/FVC	0.268
BMI	−0.12	FEV1	−0.46
Diabetes	0.073	6MWT (%)	−0.06
Pneumonia	0.239	Smoking status	−0.12

Legend: SII—The Systemic Immune-Inflammation Index; 6MWT—6 min walk test; FVC—forced vital capacity; FEV1—forced expiratory volume in the first second; BMI—Body Mass Index.

## Data Availability

The data that support the findings of this study are available from the corresponding author (A.M.) upon reasonable request. The data are not publicly available due to the principle of anonymity of the people who took part in the study. The participants signed a data confidentiality clause.

## References

[1] Asser P.A.L., Soundararajan K. (2021). The vital role of physiotherapy during COVID-19: A systematic review. Work.

[2] Filgueira T.O., Castoldi A., Santos L.E.R., de Amorim G.J., de Sousa Fernandes M.S., Anastácio W.d.L.D.N., Campos E.Z., Santos T.M., Souto F.O. (2021). The Relevance of a Physical Active Lifestyle and Physical Fitness on Immune Defense: Mitigating Disease Burden, With Focus on COVID-19 Consequences. Front. Immunol..

[3] Wang J., Jiang M., Chen X., Montaner L.J. (2020). Cytokine storm and leukocyte changes in mild versus severe SARS-CoV-2 infection: Review of 3939 COVID-19 patients in China and emerging pathogenesis and therapy concepts. J. Leukoc. Biol..

[4] Han H., Ma Q., Li C., Liu R., Zhao L., Wang W., Zhang P., Liu X., Gao G., Liu F. (2020). Profiling serum cytokines in COVID-19 patients reveals IL-6 and IL-10 are disease severity predictors. Emerg. Microbes Infect..

[5] Zhao Y., Qin L., Zhang P., Li K., Liang L., Sun J., Xu B., Dai Y., Li X., Zhang C. (2020). Longitudinal COVID-19 profiling associates IL-1RA and IL-10 with disease severity and RANTES with mild disease. J. Clin. Investig..

[6] Zanza C., Romenskaya T., Manetti A.C., Franceschi F., La Russa R., Bertozzi G., Maiese A., Savioli G., Volonnino G., Longhitano Y. (2022). Cytokine Storm in COVID-19: Immunopathogenesis and Therapy. Medicina.

[7] Zhu Z., Cai T., Fan L., Lou K., Hua X., Huang Z., Gao G. (2020). Clinical value of immune-inflammatory parameters to assess the severity of coronavirus disease 2019. Int. J. Infect. Dis..

[8] Del Valle D.M., Kim-Schulze S., Huang H.-H., Beckmann N.D., Nirenberg S., Wang B., Lavin Y., Swartz T.H., Madduri D., Stock A. (2020). An inflammatory cytokine signature predicts COVID-19 severity and survival. Nat. Med..

[9] Karimi A., Shobeiri P., Kulasinghe A., Rezaei N. (2021). Novel Systemic Inflammation Markers to Predict COVID-19 Prognosis. Front. Immunol..

[10] Velazquez S., Madurga R., Castellano J.M., Rodriguez-Pascual J., de Aguiar Diaz Obregon S.R., Jimeno S., Montero J.I., Wichner P.S.V., López-Escobar A. (2021). Hemogram-Derived Ratios as Prognostic Markers of ICU Admission in COVID-19. BMC Emerg. Med..

[11] Simadibrata D.M., Pandhita B.A.W., Ananta M.E., Tango T. (2022). Platelet-to-Lymphocyte Ratio, a Novel Biomarker to Predict the Severity of COVID-19 Patients: A Systematic Review and Meta-Analysis. J. Intensive Care Soc..

[12] Copaescu A., Smibert O., Gibson A., Phillips E.J., Trubiano J.A. (2020). The role of IL-6 and other mediators in the cytokine storm associated with SARS-CoV-2 infection. J. Allergy Clin. Immunol..

[13] Costela-Ruiz V.J., Illescas-Montes R., Puerta-Puerta J.M., Ruiz C., Melguizo-Rodríguez L. (2020). SARS-CoV-2 infection: The role of cytokines in COVID-19 disease. Cytokine Growth Factor Rev..

[14] Dhar S.K., Damodar S., Gujar S., Das M. (2021). IL-6 and IL-10 as predictors of disease severity in COVID-19 patients: Results from meta-analysis and regression. Heliyon.

[15] Ouyang W., Rutz S., Crellin N.K., Valdez P.A., Hymowitz S.G. (2011). Regulation and functions of the IL-10 family of cytokines in inflammation and disease. Annu. Rev. Immunol..

[16] Behzadi P., Behzadi E., Ranjbar R. (2016). IL-12 Family Cytokines: General Characteristics, Pathogenic Microorganisms, Receptors, and Signalling Pathways. Acta Microbiol. Immunol. Hung..

[17] Leite M.d.M., Gonzalez-Galarza F.F., da Silva B.C.C., Middleton D., dos Santos E.J.M. (2021). Predictive immunogenetic markers in COVID-19. Hum. Immunol..

[18] Kandikattu H.K., Venkateshaiah S.U., Kumar S., Mishra A. (2020). IL-15 immunotherapy is a viable strategy for COVID-19. Cytokine Growth Factor Rev..

[19] Wang W., Tang J., Wei F. (2020). Updated understanding of the outbreak of 2019 novel coronavirus (2019-nCoV) in Wuhan, China. J. Med. Virol..

[20] Iskandar A., Mayashinta D.K., Robert R., Samsu N., Endharti A.T., Widjajanto E. (2023). Correlation Between IL-8, C-Reactive Proteins (CRP) and Neutrophil to Lymphocyte Ratio (NLR) as Predictor of Mortality in COVID-19 Patients with Diabetes Mellitus Comorbidity. Int. J. Gen. Med..

[21] Chan A.S., Rout A. (2020). Use of Neutrophil-to-Lymphocyte and Platelet-to-Lymphocyte Ratios in COVID-19. J. Clin. Med. Res..

[22] Li X., Liu C., Mao Z., Xiao M., Wang L., Qi S., Zhou F. (2020). Predictive Values of Neutrophil-to-Lymphocyte Ratio on Disease Severity and Mortality in COVID-19 Patients: A Systematic Review and Meta-Analysis. Crit. Care.

[23] Ravindra R., Ramamurthy P., Aslam S.M., Kulkarni A.K.S., Ramamurthy P.S. (2022). Platelet Indices and Platelet to Lymphocyte Ratio (PLR) as Markers for Predicting COVID-19 Infection Severity. Cureus.

[24] Dymicka-Piekarska V., Dorf J., Milewska A., Łukaszyk M., Kosidło J.W., Kamińska J., Wolszczak-Biedrzycka B., Naumnik W. (2023). Neutrophil/Lymphocyte Ratio (NLR) and Lymphocyte/Monocyte Ratio (LMR)—Risk of Death Inflammatory Biomarkers in Patients with COVID-19. J. Inflamm. Res..

[25] Olivieri F., Sabbatinelli J., Bonfigli A.R., Sarzani R., Giordano P., Cherubini A., Antonicelli R., Rosati Y., Del Prete S., Di Rosa M. (2022). Routine Laboratory Parameters, Including Complete Blood Count, Predict COVID-19 in-Hospital Mortality in Geriatric Patients. Mech. Ageing Dev..

[26] Righetti R.F., Onoue M.A., Politi F.V.A., Teixeira D.T., de Souza P.N., Kondo C.S., Moderno E.V., Moraes I.G., Maida A.L.V., Pastore L. (2020). Physiotherapy Care of Patients with Coronavirus Disease 2019 (COVID-19)—A Brazilian Experience. Clinics.

[27] Order of the President of the National Health Fund No. 172/2021/DSOZ of 18 October 2021. https://baw.nfz.gov.pl/NFZ/tabBrowser/mainPage.

[28] Klok F.A., Boon G.J., Barco S., Endres M., Geelhoed J.M., Knauss S., Rezek S.A., Spruit M.A., Vehreschild J., Siegerink B. (2020). The Post-COVID-19 Functional Status scale: A tool to measure functional status over time after COVID-19. Eur. Respir. J..

[29] Paternostro-Sluga T., Grim-Stieger M., Posch M., Schuhfried O., Vacariu G., Mittermaier C., Bittner C., Fialka-Moser V. (2008). Reliability and validity of the Medical Research Council (MRC) scale and a modified scale for testing muscle strength in patients with radial palsy. J. Rehabil. Med..

[30] Minakata Y., Hayata A., Matsunaga K., Nakanishi M., Yamamoto N. (2016). Differences in physical activity according to mMRC grade in patients with COPD. Int. J. Chronic Obstr. Pulm. Dis..

[31] Graham B.L., Steenbruggen I., Miller M.R., Barjaktarevic I.Z., Cooper B.G., Hall G.L., Hallstrand T.S., Kaminsky D.A., McCarthy K., McCormack M.C. (2019). Standardization of Spirometry 2019 Update. An Official American Thoracic Society and European Respiratory Society Technical Statement. Am. J. Respir. Crit. Care Med..

[32] Singh S.J., Puhan M.A., Andrianopoulos V., Hernandes N.A., Mitchell K.E., Hill C.J., Lee A.L., Camillo C.A., Troosters T., Spruit M.A. (2014). An official systematic review of the European Respiratory Society/American Thoracic Society: Measurement properties of field walking tests in chronic respiratory disease. Eur. Respir. J..

[33] Mandaliya H., Jones M., Oldmeadow C., Nordman I.I.C. (2019). Prognostic biomarkers in stage IV non-small cell lung cancer (NSCLC): Neutrophil to lymphocyte ratio (NLR), lymphocyte to monocyte ratio (LMR), platelet to lymphocyte ratio (PLR) and advanced lung cancer inflammation index (ALI). Transl. Lung Cancer Res..

[34] Wang R.-H., Wen W.-X., Jiang Z.-P., Du Z.-P., Ma Z.-H., Lu A.-L., Li H.-P., Yuan F., Wu S.-B., Guo J.-W. (2023). The clinical value of neutrophil-to-lymphocyte ratio (NLR), systemic immune-inflammation index (SII), platelet-to-lymphocyte ratio (PLR) and systemic inflammation response index (SIRI) for predicting the occurrence and severity of pneumonia in patients with intracerebral hemorrhage. Front. Immunol..

[35] Ali A.M., Rostam H.M., Fatah M.H., Noori C.M., Ali K.M., Tawfeeq H.M. (2022). Serum troponin, D-dimer, and CRP level in severe coronavirus (COVID-19) patients. Immun. Inflamm. Dis..

[36] Chi Y., Ge Y., Wu B., Zhang W., Wu T., Wen T., Liu J., Guo X., Huang C., Jiao Y. (2020). Serum cytokine and chemokine profile in relation to the severity of coronavirus disease 2019 in China. J. Infect. Dis..

[37] Jia F., Wang G., Xu J., Long J., Deng F., Jiang W. (2021). Role of tumor necrosis factor-α in the mortality of hospitalized patients with severe and critical COVID-19 pneumonia. Aging.

[38] Kgatle M.M., Lawal I.O., Mashabela G., Boshomane T.M.G., Koatale P.C., Mahasha P.W., Ndlovu H., Vorster M., Rodrigues H.G., Zeevaart J.R. (2021). COVID-19 Is a Multi-Organ Aggressor: Epigenetic and Clinical Marks. Front. Immunol..

[39] Ji Y., Li M., Chang M., Liu R., Qiu J., Wang K., Deng C., Shen Y., Zhu J., Wang W. (2022). Inflammation: Roles in Skeletal Muscle Atrophy. Antioxidants.

[40] Ma W., Xu T., Wang Y., Wu C., Wang L., Yang X., Sun H. (2018). The role of inflammatory factors in skeletal muscle injury. Biotarget.

[41] Pahlavani H.A. (2022). Exercise Therapy for People with Sarcopenic Obesity: Myokines and Adipokines as Effective Actors. Front. Endocrinol..

[42] Sirayder U., Inal-Ince D., Kepenek-Varol B., Acik C. (2022). Long-Term Characteristics of Severe COVID-19: Respiratory Function, Functional Capacity, and Quality of Life. Int. J. Environ. Res. Public Health.

[43] Chen D., Xie J., Fiskesund R., Dong W., Liang X., Lv J., Jin X., Liu J., Mo S., Zhang T. (2018). Chloroquine modulates antitumor immune response by resetting tumor-associated macrophages toward M1 phenotype. Nat. Commun..

[44] Chua R.L., Lukassen S., Trump S., Hennig B.P., Wendisch D., Pott F., Debnath O., Thürmann L., Kurth F., Völker M.T. (2020). COVID-19 severity correlates with airway epithelium–immune cell interactions identified by single-cell analysis. Nat. Biotechnol..

[45] Meisen W.H., Wohleb E.S., Jaime-Ramirez A.C., Bolyard C., Yoo J.Y., Russell L., Hardcastle J., Dubin S., Muili K., Yu J. (2015). The Impact of Macrophage- and Mi-croglia-Secreted TNFα on Oncolytic HSV-1 Therapy in the Glioblastoma Tumor Microenvironment. Clin. Cancer Res..

[46] Lv J., Wang Z., Qu Y., Zhu H., Zhu Q., Tong W., Bao L., Lv Q., Cong J., Li D. (2021). Distinct uptake, amplification, and release of SARS-CoV-2 by M1 and M2 alveolar macrophages. Cell Discov..

